# How digital mismatch leads to digital burnout among grassroots public servants in China: the moderating role of leadership care and organizational incentives

**DOI:** 10.3389/fpubh.2026.1700296

**Published:** 2026-04-01

**Authors:** Hui Li, Zhenjing Pang

**Affiliations:** 1 Chengdu Vocational & Technical College of Industry, Chengdu, China; 2 School of Public Administration, Sichuan University, Chengdu, China

**Keywords:** digital burnout, digital mismatch, leadership care, occupational health, organizational incentives, public servants, technology identity, work stress

## Abstract

**Background:**

Digital burnout is an increasingly prominent mental health issue in the modern digitalized workplace. As promoters, implementers, and experiencers of digital transformation, grassroots public servants face heightened occupational health risks in technology-driven environments. This study aims to explore the formative and buffering mechanisms of digital burnout among them, providing empirical evidence for maintaining the well-being of the public servant workforce and constructing a sustainable digital work environment.

**Methods:**

A questionnaire survey was conducted using convenience sampling and snowball sampling methods. A total of 656 valid questionnaires were collected from three cities in China (Shanghai, Wuhan, and Chengdu), representing the eastern, central, and western regions of the country, respectively. The data were analyzed using Confirmatory Factor Analysis, Structural Equation Modeling, and Regression Analysis to test the proposed hypotheses.

**Results:**

Digital mismatch was identified as a critical antecedent to digital burnout. Specifically: (1) Digital suspension and digital overload positively predicted work stress, which in turn significantly exacerbated digital burnout; (2) Digital suspension and the digital divide had negative impact on technology identity, which effectively mitigated digital burnout; (3) Leadership care and organizational incentives played a significant negative moderating role in the relationship between digital overload and work stress, indicating that organizational support is a crucial buffering factor.

**Conclusion:**

The results indicate that digital mismatch affects digital burnout through dual mechanisms of “increased work stress” and “reduced technology identity,” while confirming the moderating effects of leadership care and organizational incentives in certain paths. These findings suggest that alleviating digital burnout among employees requires focused efforts on digital suspension, the digital divide, and digital overload, by establishing an occupational health protection system encompassing technological, organizational, and individual dimensions.

## 1 Introduction

The evolution of technology in the digital age is constantly reshaping work patterns and organizational models. With the deep application of digital technologies, such as artificial intelligence and cloud computing, workplaces are increasingly characterized by a high level of digitization and interconnectivity (1). Against this backdrop, the work patterns in the public service sector have also undergone significant changes (2), with digital government platforms, office automation systems, and mobile applications becoming core tools in the daily work of grassroots public servants (3). Although these technological tools are designed to enhance work efficiency and service effectiveness, their widespread adoption may also introduce new occupational health challenges (4), particularly digital burnout—a phenomenon caused by systemic mismatches between technological functions, organizational demands, and individual capabilities (5). The occupational health risks associated with the use of digital technologies have become a critical issue for employee well-being and organizational sustainability in digital work environments.

Digital burnout, as a unique psychological occupational health issue specific to the digital age, differs from traditional occupational burnout (6) and technostress (7). Specifically, it refers to a series of psychological reactions resulting from prolonged interaction with digital technologies, including emotional exhaustion, depersonalization, and reduced personal accomplishment (8). Therefore, digital burnout is considered a unique occupational health risk associated with digital technology use. However, existing research has primarily focused on digital burnout among the general public (9–11), yet it has largely overlooked a critical occupational group—grassroots public servants. This group requires frequent and intensive use of various digital platforms in their daily work. They are not only heavy users of digital technologies but also core implementers and facilitators of digital transformation (12). This multifaceted role exposes them to unique digital adaptation challenges and work-related stress, making them an ideal sample group for studying occupational health risks in the digital age.

Based on this understanding, this study focuses on Chinese grassroots public servants as the research subjects to examine the impact of digital mismatch (comprising digital suspension, digital divide, and digital overload) on digital burnout. By constructing and validating a dual-path theoretical model, this study systematically examines the underlying mechanisms through which digital mismatch affects digital burnout via two parallel mechanisms: increased work stress (13) and reduced technology identity (14). Furthermore, we examine the moderating effects of leadership care and organizational incentives on the relationship between the digital environment and psychological responses. The findings not only contribute to a deeper understanding of emerging occupational health challenges in the digital age and their underlying mechanisms, but also provide robust empirical evidence for enhancing organizational sustainability and employee psychological well-being. These findings offer significant implications for developing mental health promotion strategies in various digital work environments.

## 2 Literature review

### 2.1 Digital burnout

With the deepening integration of digital technology into the bureaucratic framework, digital burnout has gradually attracted scholarly attention. At present, relevant research is still in its infancy, yet the concept is closely related to such theoretical constructs as occupational burnout and digital dysfunction.

On the one hand, in terms of conceptual relevance, digital burnout originates from occupational burnout. Occupational burnout was first proposed by Freudenberger (15), and is generally defined as a state of physical and mental exhaustion experienced by individuals in the workplace when confronted with excessive work demands (16). It represents an extreme response to prolonged stressful working environments, and develops into a clinical chronic syndrome under continuous accumulation. Occupational burnout is typically manifested along three dimensions: emotional exhaustion, cynicism, and reduced professional efficacy. The World Health Organization (WHO) identifies three key features of occupational burnout: energy depletion or exhaustion, increased mental distance from one’s job, or feelings of negativism or cynicism related to one’s job, and reduced professional efficacy (17). Current empirical evidence in this field remains largely concentrated on high-stress occupational groups, such as teachers who routinely engage in emotional labor (18) and healthcare workers operating in high-pressure shift environments (19).

With the rapid development and extensive application of digital technology across various sectors, digital technology has become intertwined with occupational burnout, bringing the concept of digital burnout into the scope of academic research. This new form of burnout manifests through digital aging, digital deprivation, and emotional exhaustion resulting from prolonged exposure to digital environments (20). Its negative impacts have been documented across various occupational groups: among healthcare professionals, excessive use of digital tools in the workplace is associated with increased workload, heightened security risks, and information quality issues (21); among teachers, burnout triggered by digital environments directly reduces job satisfaction and impacts students’ academic performance (22); and among online gig workers, technology overload contributes to burnout and diminished creative performance (23). However, despite being a high-exposure group in digital work environments, the psychological mechanisms and formation mechanisms of digital burnout among public servants have not yet been systematically studied.

From a theoretical perspective, technostress, digital burnout, and occupational burnout form a spectrum of causally linked and interrelated concepts. Technostress is the root cause, describing the negative experiences arising from information technology itself as a stressor (9). Digital burnout is a specific intensification and manifestation of technostress in the hyper-connected era, referring explicitly to the emotional numbness, cognitive overload, and avoidance behaviors triggered by “always-on” culture, social media, and digital interactions. It can be seen as a state of mental and physical exhaustion in a specific domain resulting from the accumulation of technostress. Occupational burnout, on the other hand, is a broader, classic psychological syndrome whose core focus is the work context itself (24). In short, technostress is the cause, digital burnout is its effect and manifestation in the digital realm, and together they constitute key drivers that trigger or exacerbate modern occupational burnout. Thus, digital burnout acts as an amplifier, channeling and integrating technostress into an individual’s professional life, ultimately potentially manifesting as the classic symptoms of occupational burnout: exhaustion, cynicism, and reduced efficacy toward work itself. Understanding this chain is crucial for diagnosing and intervening in the health of organizations and individuals in the digital age.

Overall, digital burnout is an emerging concept shaped by the broader context of occupational burnout and the pressures of the digital era. It is not confined to occupational contexts; rather, it can be generally applied to explain the burnout-related negative psychological states experienced by diverse individuals in a digitalized society, including irritability, cognitive impairment, emotional fatigue, anxiety, and stress. Such states exert extensive impacts on interpersonal relationships, work performance, and social activities, such as increased anxiety, exhaustion, apathy, and reduced interest in life or work.

Against the current backdrop of digital governance, an increasing number of grassroots civil servants are suffering from digital burnout, which has produced notable negative effects and merits considerable attention. The digital burnout of grassroots civil servants focused on in this study is defined as follows: in digital governance scenarios, the negative psychological and behavioral states of grassroots civil servants caused by excessive administrative burdens and other factors stemming from the application of digital technology, including behavioral burnout, emotional exhaustion, and meaning erosion. As a specific manifestation of occupational burnout in the digital domain, it includes feelings of powerlessness toward the iteration of digital technology, dissatisfaction with digital tasks encroaching on personal life, and chronic fatigue under long-term digital pressure. For example, some grassroots cadres feel overwhelmed, unconfident, and depressed when forced to invest considerable energy in learning and operating complex and redundant digital systems, which triggers passive work attitudes and resistant behaviors and seriously hinders the effective operation of digital government.

### 2.2 Digital mismatch (digital suspension, digital divide, and digital overload)

Digital mismatch refers to the structural imbalance between technological functions, organizational demands, and individual capabilities within digital work environments (25). This phenomenon stems from the contradiction between organizations’ operational needs in dynamic environments and the multidimensional coordination required for digital transformation (26). Excessive organizational reliance on digital technologies may lead to passive-aggressive behaviors among employees, subsequently triggering digital burnout and psychological resistance (27). Employee psychological barriers are a critical factor in the failure of organizational digital transformation (28). As the research perspective shifts from purely technological approaches to behavioral psychology viewpoints, growing evidence has uncovered potential occupational health risk factors in digital work environments (29, 30). Based on this theoretical framework, this paper categorizes digital mismatch into three distinct types: digital suspension, digital divide, and digital overload.

Digital suspension manifests as a mismatch between technological functions and organizational demands. This occurs when the design and application of technological tools become disconnected from actual workflows and organizational structures, leading to decreased efficiency and wasted resources (31). For instance, complex digital platforms may compel employees to spend excessive time on data entry and procedural operations, triggering work burnout and perceived inefficacy (32). While existing research often focuses on the impact of digital suspension on productivity, its mechanisms affecting employee well-being and mental health remain underexplored.

The digital divide manifests as a mismatch between technological functions and individual capabilities. When digital systems’ complexity and operational demands exceed employees’ cognitive and skill levels, it is associated with intense technological anxiety and avoidance behaviors (33). For instance, faced with complex online approval systems, some employees may engage in passive resistance due to fear of operational errors, ultimately impairing work efficiency (34). This indicates that existing digital training and support systems often overlook employee capability disparities and lack specificity.

Digital overload manifests as a mismatch between organizational demands and individual capabilities. When organizations impose excessive or unreasonable digital expectations exceeding employees’ psychological resilience and work efficiency, severe mental health risks emerge (35). Algorithm-driven incentive and penalty mechanisms may sustain employees in high-pressure work states, closely linked to increased risks of depression, anxiety, and decision fatigue (36). Further research is needed in this area, particularly regarding the long-term health impacts of chronic digital overload on employees.

In summary, although existing studies have separately examined the three types of digital mismatch and their negative effects on occupational health, several limitations remain. First, most analyses focus on a single type in isolation, lacking a holistic examination within a triadic model framework. Second, empirical research based on large-scale samples remains insufficient. Therefore, this study will empirically examine how these three types of digital mismatch influence digital burnout through work stress and technology identity, using structural equation modeling. This approach will provide more comprehensive theoretical and practical references for safeguarding employee well-being in the digital age.

### 2.3 The influencing factors of digital burnout

The rapid development of digital technology has contributed to higher quality and greater efficiency in grassroots governance. However, it has also led to adaptive problems between new digital elements and traditional institutional structures, triggering negative consequences such as intelligent bureaucracy, digital formalism, digital burnout, and other related issues (37). Reviewing existing studies, scholars have conducted relatively limited research on digital burnout. Most studies remain focused on the increased burdens caused by digitalization, with preliminary explorations into the influencing factors of digital burden, mainly from the perspectives of bureaucratic systems, technological pressure, and individual information cognition (38). Meanwhile, a certain body of research on digital burnout has emerged, which identifies multiple influencing factors as follows.

First, at the institutional environment level, the soaring compliance costs and psychological costs induced by institutional design and governance models directly intensify work pressure (39). The institutional roots of administrative burden constitute the core cause of digital burnout among grassroots civil servants. To begin with, under the dual pressure of the pressure-driven system and the target responsibility system, characterized by “layered escalation” and “one-vote veto,” together with rigid assessment requirements under the target responsibility system, grassroots authorities are trapped in formalistic pitfalls such as “data competition” and “excessive record-keeping,” further increasing administrative burdens. Second, due to the short-term and mandatory promotion of campaign-style governance, digital transformation has been designated a “central task” and rolled out rapidly. Indicators including the registration rate of government apps and news likes have been incorporated into performance assessment, requiring the completion of digital construction within a short period. The proliferation of digital tools has left grassroots cadres overwhelmed, with continuous accumulation of psychological pressure. Third, the timeless and spaceless nature of digital technology has blurred the boundary between work and life for grassroots civil servants, exposing them to pressures such as invisible overtime and real-time accountability. For instance, they have to respond to work group messages and submit data after work, leading to a sense of deprivation in personal life.

Second, at the level of technological characteristics, digital technology has deviated from its intended functions in actual grassroots governance applications for various reasons (40). Technological triggers of digital dysfunction have turned technological empowerment into burden enhancement, raising the learning costs and operational barriers for grassroots civil servants. On the one hand, technological functions are subject to internal competition and redundancy. Digital tools are developed excessively for competitive advantages, ignoring actual grassroots demands and resulting in overly complex and redundant functions. For example, various government apps and mini-programs are repeatedly developed, lacking compatibility and making data migration difficult. Grassroots cadres have to spend considerable time learning the operational logic of different tools. The intensity and duration of digital device use can directly threaten users’ mental health, increasing stress and anxiety and leading to digital burnout (41). Some technologies even fall into the trap of pseudo digitalization, which merely adopts a digital appearance without solving practical problems. Further technological pressure, decreased job satisfaction, lack of work interest, and cognitive biases also contribute to digital burnout (42). On the other hand, the absence of unified technological standards and supervision has led to the prevalence of “digital formalism at the fingertips”, such as repeated completion of electronic forms and inconsistent data formats. Grassroots cadres have to spend extra energy on verification and adjustment, increasing compliance costs and ineffective burdens.

Finally, at the level of individual cognition and literacy, insufficient digital literacy and unbalanced psychological status among grassroots civil servants more or less amplify the burdens imposed by technology and institutions (43). On the one hand, digital literacy varies greatly among grassroots civil servants, resulting in a digital divide across regions and administrative levels. On the other hand, some grassroots civil servants show low technological identity and psychological resistance. They lack sufficient understanding of the value of digital technology and hold resistant attitudes such as inability to learn, incomprehension, and reluctance to adopt new tools. Young cadres resent meaningless digital tasks, while senior cadres experience frustration due to inadequate capability. The lack of technological identity directly reduces initiative in digital work and triggers passive work attitudes. In addition, some scholars argue that physical and mental health and economic conditions at the individual level also serve as important influencing factors (44).

### 2.4 The alleviation paths of digital burnout

At present, existing studies focus on addressing the increasingly prominent digital burnout among grassroots civil servants through such approaches as knowledge adjustment, value adjustment, incentive adjustment, and environmental improvement.

First, the knowledge adjustment approach. Knowledge adjustment constitutes the cognitive basis for alleviating digital burnout. Its core function lies in bridging the digital literacy gap, relieving anxiety caused by technological unfamiliarity, and reconstructing a positive interactive relationship between grassroots civil servants and digital technologies (8). Digital burnout among grassroots civil servants often stems from insufficient digital knowledge reserves and unskilled technical operations, which lead to frequent obstacles in data entry, system maintenance, online services, and other tasks, thereby generating frustration and powerlessness. Digital literacy is a basic skill requirement for grassroots cadres to adapt to grassroots digital governance. It mainly refers to the ability of grassroots cadres to understand digital devices and operating software, use digital tools, and acquire basic knowledge of grassroots digital governance. It primarily includes two components: digital acquisition and digital socialization. Grassroots cadres are required to not only proficiently use various digital hardware and software for information searching and accurately identify diverse digital content, but also utilize digital tools or platforms for information communication, transmission, and sharing, so as to realize the efficient transfer of digital information across time and space.

Second, the value adjustment approach. Value adjustment serves as the intrinsic core of relieving digital burnout. Its key function is to reconstruct the perception of the meaning of digital work and awaken grassroots civil servants’ professional mission and value identity (11). The instrumental nature of digital technologies tends to trap grassroots civil servants in the cognitive misunderstanding of mechanical data processing, making them ignore the core value of digital government affairs in improving service efficiency and governance effectiveness, and thus triggering a sense of meaningless burnout. In terms of ideology, grassroots cadres should gradually shift from traditional empirical thinking to data-driven scientific decision-making awareness, establish a sense of the times, and fully recognize that enhancing digital literacy is a practical requirement to keep pace with the times. They should deeply understand and actively implement the national big data development strategy, proactively adapt to the requirements of grassroots digital governance in the new era, and gradually overcome resistance and fear toward the digital era.

Third, the incentive adjustment approach. Incentive adjustment acts as the external driver for mitigating digital burnout. Its core function is to strengthen positive behavioral feedback on grassroots civil servants’ digital work and improve their sense of achievement and initiative through the design of a scientific incentive mechanism (45). Organizational factors are important triggers of burnout (46). For employees in the public sector, promotion serves as a major incentive and a source of public service motivation. In other words, the key to organizational governance lies in people; focusing on people requires strengthening incentives to tap their potential and enthusiasm (47).

Fourth, the environmental adjustment approach. Environmental adjustment provides the supportive guarantee for relieving digital burnout. Its key function is to reduce the objective workload and create an inclusive working atmosphere by optimizing the technical and organizational environments for digital work (48). From the perspective of the technical environment, some grassroots digital systems suffer from cumbersome interfaces, redundant functions, and incompatible data, forcing grassroots civil servants to enter data repeatedly and switch between systems, which greatly increases time consumption and energy expenditure. Environmental adjustment reduces the physical burden of digital work by promoting lightweight transformation, functional integration, and data sharing of digital systems, simplifying operational processes, and eliminating ineffective labor. From the perspective of the organizational environment, organizational behavior and management practices are also closely associated with burnout. For instance, excessive red tape, discipline, and supervision in bureaucratic organizations are more likely to induce burnout (7). Targeted measures include establishing a support mechanism for digital work and implementing a flexible working system to provide technical support and physical and mental recovery space for grassroots civil servants. Meanwhile, fostering an organizational atmosphere of “error tolerance and correction” can alleviate anxiety in digital work and provide a stable and friendly environmental guarantee for grassroots civil servants to carry out digital tasks.

In addition, Harmon and Duffy proposed digital disconnection as a coping strategy, which refers to reducing or discontinuing dependence on digital devices and technologies (49). However, this approach faces considerable operational challenges. Some scholars have attempted to explore pathways to mitigate digital burnout from three dimensions: “human,” “technology,” and “human-technology relationship” (50). Others have put forward solutions to digital burnout from four aspects: the technological dimension, the temporal dimension, the teamwork dimension (team care and information sharing), and the process dimension (51). Nevertheless, these solutions still suffer from limited applicability in the context of grassroots governance.

Existing research has produced inspiring and valuable findings, which are of great significance for enriching the theory and practice of digital burnout among grassroots civil servants. Nevertheless, the following limitations remain and require further investigation. In terms of conceptual definition, the connotation of digital burnout is mostly an extension of the concept of job burnout, lacking a precise deconstruction based on the particularity of the digital context. In particular, it has not sufficiently integrated the professional attributes, work scenarios and institutional constraints of grassroots civil servants, nor has it formed a dedicated conceptual framework and dimensional division suitable for the context of grassroots civil servants. As a result, the contextual relevance of the concept and the accuracy of its measurement are insufficient, and the conceptual connotation needs to be further refined and elevated.

Regarding the exploration of formation mechanisms, existing studies mostly adopt a single perspective, such as functional defects of technology itself, shortcomings in individuals’ digital literacy, or subjective cognitive biases. They ignore the core contextual contradiction of maladjustment between “human” and “technology” after digital technology is embedded in grassroots governance, and fail to reveal the formation logic of digital burnout from the dynamic relationship of human-technology interaction. Therefore, the perspective on its formation mechanism urgently needs to be supplemented with contextual factors.

In the research on intervention strategies, existing findings mostly focus on individual-level strategies such as improving digital literacy and psychological adjustment, with insufficient attention paid to organizational environmental factors. However, organizational factors are crucial in reconciling human-technology mismatches and thereby alleviating digital burnout among grassroots civil servants. Existing studies have not fully explored the moderating role of organizational support in mitigating digital burnout, leading to a lack of systematicness and operability in intervention approaches.

Overall, existing research has not fully addressed the contextual particularity and core contradictions of digital burnout among grassroots civil servants. This study strives to remedy the above deficiencies: on the basis of accurately defining the connotation and dimensions of digital burnout adapted to the scenario of grassroots civil servants, this paper systematically reveals the formation mechanism of digital burnout from the core perspective of digital mismatch. Furthermore, it introduces organizational support and leadership care as moderating variables to explore targeted and feasible intervention strategies, so as to provide useful references for the deepening of relevant theoretical research and the optimization of grassroots governance practice.

## 3 Conceptual model and hypotheses

### 3.1 Conceptual model

Based on the Job Demands-Resources model (JD-R Model) and the Stimuli-Organism-Response theory (SOR), this study constructs a “Digital Mismatch-Work Stress/Technology Identity-Digital Burnout” structural model to explain how Digital Mismatch affects Digital Burnout, as well as the moderating roles of Leadership Care and Organizational Incentives within it.

This model follows the causal chain of the SOR theory (Stimuli-Organism-Response) and integrates the dual-process mechanism of the Job Demands-Resources model, thereby forming a theoretical framework for understanding how digital mismatch affects digital burnout (52). According to the SOR theory, Digital Suspension, Digital Divide, and Digital Overload can be defined as external stimuli, representing core job demands in the digital environment. In the middle, Work Stress and Technology Identity constitute the internal psychological state (Organism) formed after individuals receive these stimuli. Ultimately, Digital Burnout represents the psychological response (Response) resulting from long-term imbalance in individuals. Meanwhile, according to the JD-R model, it is further clarified that digital demands, such as information overload, skills gap, and attention fragmentation, serve as the starting point of the health impairment process by continuously consuming physical and psychological resources (53). Leadership Care and Organizational Incentives, as key job resources, can not only directly buffer the pressure caused by digital demands but also moderate the transmission intensity of the “Stimuli-Organism-Response” pathway, reflecting the dual role of job resources in motivation and health protection within the JD-R model (54).

**Figure 1:**
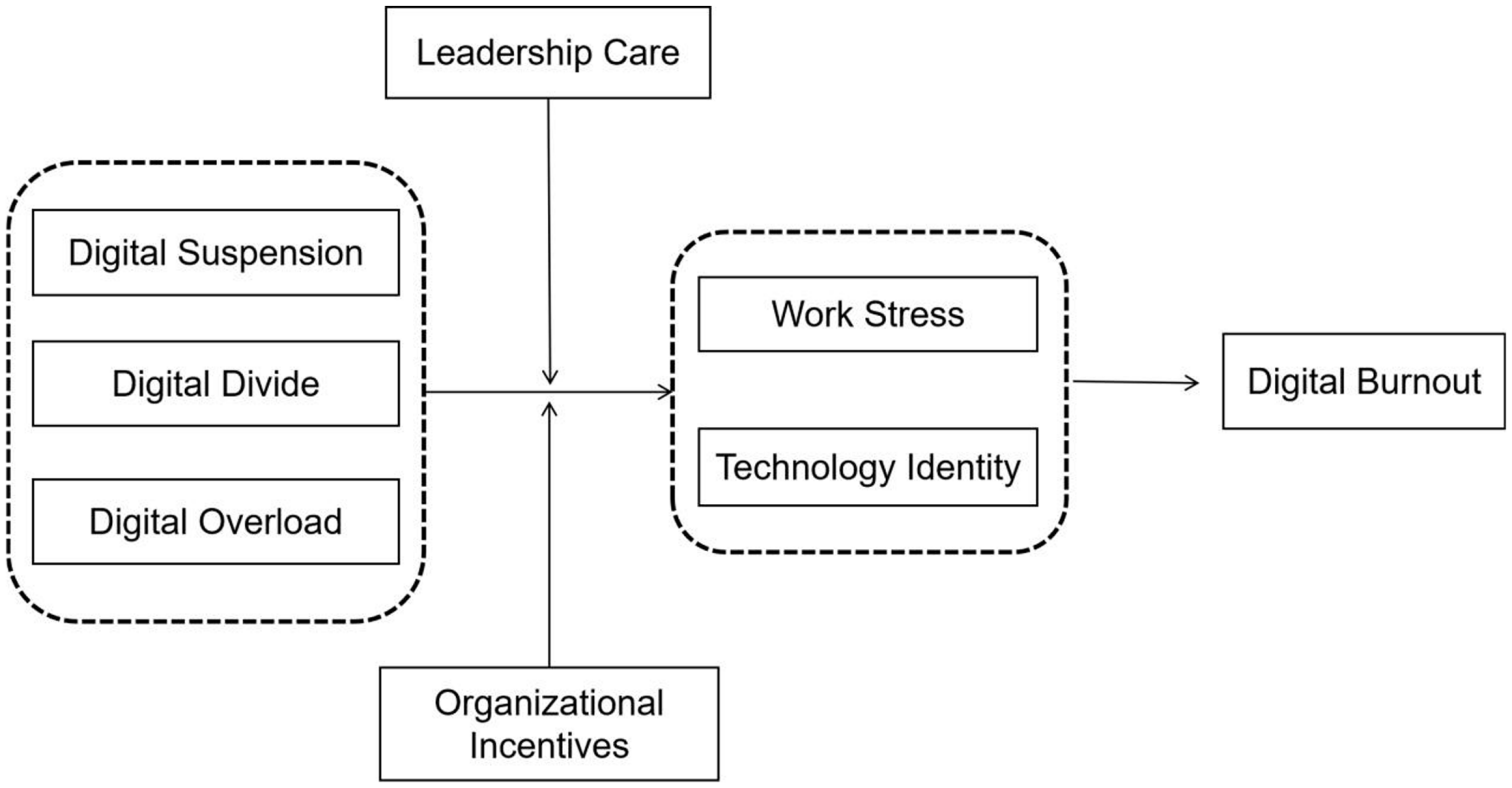
Conceptual model.

This model delineates the complete formation pathway of digital burnout and its corresponding intervention logic. First, digital mismatch, as the core source of stimuli, continuously acts upon individuals. Specifically, Digital Overload depletes cognitive resources through excessive information and tool inputs, Digital Divide exacerbates role pressure and diminishes self-efficacy due to skill gaps, and Digital Suspension increases cognitive load via attention fragmentation. Collectively, these three factors constitute high-intensity job demands. Second, these demands are mediated by individual psychological states (the organism), consequently leading to digital burnout. Work Stress, as a direct manifestation of physical and psychological tension, represents a core expression of the health impairment process. Technology Identity, through the degree of an individual’s identification with and integration of digital technology, in turn shapes their susceptibility to digital burnout. When digital mismatch persistently exceeds an individual’s resource threshold, and their psychological state remains in long-term disequilibrium, Digital Burnout is triggered, characterized by emotional exhaustion, depersonalization, and reduced personal accomplishment. Furthermore, Leadership Care and Organizational Incentives serve as key moderating variables. By reducing perceived stress through leadership support and enhancing digital competence and technology identity through organizational incentives, they effectively buffer the transmission from digital mismatch to digital burnout, thereby providing a clear pathway for intervention (see Figure 1).

### 3.2 Hypotheses

#### 3.2.1 The relationship between digital mismatch and work stress

As a typical alienation phenomenon in the digital transformation of grassroots governance, digital suspension mainly refers to the disembedded state between digital technology and actual governance needs, manifesting in specific forms such as technical tools floating above grassroots work, exceeding the capacity of governance subjects, and failing to align with the demands of the public (55). The concept reveals the potential “acclimatization failure” that digital technology may encounter when embedded in grassroots governance, when digital tools are applied simplistically and coercively in grassroots practice without fully adapting to the complexity and differentiated needs of grassroots work, technology is alienated from an enabling instrument into an additional source of burden. For grassroots civil servants at the forefront of governance, digital suspension constitutes a persistent stressor in their work environment.

Existing empirical studies have provided preliminary evidence for this logical chain. Research shows that perceived technology overload has a significant positive effect on grassroots civil servants’ perceived digital burden, with perceived pressure-based institutional environment playing a positive moderating role—i.e., the greater the pressure from higher-level assessments, the stronger the impact of technology overload on burden perception (56–58). Other studies have found that digital suspension directly increases the digital burden on governance subjects, trapping grassroots cadres in a “busy but futile” involutionary state (59). These findings collectively point to a basic judgment: as a typical manifestation of failed technology embedding, digital suspension continuously elevates the work stress level of grassroots civil servants through pathways such as increasing workload, intensifying skill pressure, and weakening work autonomy. Accordingly, the following research hypothesis can be proposed:

*H1:* Digital suspension has a significant positive impact on the work stress of grassroots civil servants.

In the context of the digital transformation of grassroots governance, the digital divide primarily refers to the differentiation among grassroots civil servants arising from disparities in digital skills, technological application capabilities, and information acquisition and processing levels (60). The relative lack of digital technology application capabilities among this group constitutes a persistent source of stress in their work environment.

First, the digital divide directly leads to inequality in access to information and resources, and this information lag exacerbates time pressure and role ambiguity at work. Second, the digital divide gives rise to inequality in governance participation opportunities: those with insufficient digital skills struggle to fully participate in decision-making, and their right to participate in decision-making and voice are substantially weakened (61). This sense of disenfranchisement further amplifies the perception of work pressure. Furthermore, the digital divide is closely linked to career development opportunities. With the deepening application of digital technology in grassroots governance, demand for talent proficient in digital technologies has grown significantly for many key positions (62). Digitally vulnerable groups are placed at a disadvantage in internal job competition and promotion, and the anxiety stemming from restricted career development becomes a significant source of psychological pressure.

The digital divide continuously elevates the work pressure of grassroots civil servants through pathways such as delayed information acquisition, restricted participation opportunities, and blocked career development. When individuals with insufficient digital skills are forced to operate in a highly digitalized governance context, the competence gap between their abilities and job requirements translates into a persistent state of physical and mental tension. Accordingly, the following research hypothesis can be proposed:

*H2:* The digital divide has a significant positive impact on the work stress of grassroots civil servants.

Digital overload refers to a state of excessive burden experienced by individuals in the context of digital transformation, where the volume of information, frequency of communication, and system operation requirements resulting from the overuse of digital technology tools exceed their processing capacity (63). Digital overload has become a prevalent new work stressor faced by grassroots civil servants.

First, digital overload directly leads to the continuous depletion of cognitive resources. Grassroots civil servants need to simultaneously process information from multiple channels, respond to communication demands from various parties, and frequently switch between multiple systems. This multitasking mode continuously consumes attentional resources, resulting in the accumulation of cognitive load (64). According to the Conservation of Resources (COR) Theory, when work requirements continuously deplete an individual’s limited physical and mental resources without sufficient resource replenishment, a stress response is triggered (65). Second, digital overload blurs the boundary between work and non-work through an “always-on” work model, exacerbating work-time intrusion and the lack of recovery experience, thereby amplifying the perception of stress. Furthermore, digital overload overlaps with the pressure-based institutional environment, forming a “digital escalation” effect (66). Under the pressure of assessments from higher authorities, digital indicators have been incorporated into the performance appraisal system. Grassroots civil servants have to invest more time in digital system operations to meet assessment requirements, and this work method of “digitization for the sake of digitization” further increases the work load.

As a typical manifestation of the negative functions of technology in digital governance, digital overload continuously elevates the work stress level of grassroots civil servants through multiple pathways, such as cognitive resource depletion, blurred work boundaries, and digital assessment escalation. When information overload, communication overload, and system overload exceed an individual’s coping capacity and overlap with the pressure-based institutional environment, a persistent state of physical and mental tension is formed. Accordingly, the following research hypothesis can be proposed:

*H3:* Digital overload has a significant positive impact on the work stress of grassroots civil servants.

#### 3.2.2 The relationship between digital mismatch and technology identity

Technology identity refers to the degree of value recognition, emotional acceptance, and behavioral integration of an individual towards technological tools formed through continuous interaction with digital technology, reflecting the depth to which an individual internalizes technology into their work methods and professional identity (67). In the digital transformation of grassroots governance, technology identity serves as the psychological foundation for the in-depth integration of digital technology and governance subjects, directly affecting grassroots civil servants’ acceptance of technology, willingness to use it, and ability to apply it innovatively.

Digital suspension refers to a state of suspension formed when digital technology is embedded in the field of grassroots governance and fails to deeply align with the grassroots work context, the capabilities of governance subjects, and the actual needs of the masses. When digital tools are suspended above grassroots work, divorced from the actual capabilities of grassroots cadres, and inconsistent with the demands of the masses, technology is alienated from an enabling tool into an “alien force” external to governance subjects (68). The accumulation of such alienating experiences will fundamentally shake grassroots civil servants’ value recognition and psychological acceptance of digital technology.

From a theoretical perspective, the negative impact of digital suspension on grassroots civil servants’ technology identity has a multi-dimensional generation logic. Firstly, digital suspension undermines the perception of technology’s usefulness through the disconnection between “tools and needs”. Some scholars have pointed out that many digital governance tools fail to fully consider the actual needs of grassroots. When grassroots civil servants repeatedly experience the misalignment between digital tools and the needs of their own work, they will form a cognitive schema of “technology being useless” (69). The weakening of the perception of technology’s usefulness directly erodes the cognitive foundation of technology identity. Secondly, digital suspension impairs the perception of technology’s ease of use. When the complexity of technology operation exceeds the skill reserve of grassroots civil servants, or the speed of system iteration exceeds an individual’s learning and adaptation ability, continuous frustrating technological experiences will strengthen the negative cognition of “technology being difficult to use”, thereby reducing an individual’s willingness to accept technology and emotional attachment to it (70). Thirdly, digital suspension is likely to trigger a crisis of value identity. When grassroots civil servants feel that digital technology not only fails to serve their core work mission but also becomes an alien force that squeezes the time for substantive work, the cadres’ sense of identity and belonging to the technological system will inevitably be damaged (71).

As the core manifestation of technological embedding failure, digital suspension systematically impairs grassroots civil servants’ recognition of digital technology through multiple paths, such as undermining the perception of technology’s usefulness, weakening the experience of technology’s ease of use, and triggering a crisis of technological value identity. So the following research hypothesis can be proposed:

*H4:* Digital suspension has a significant negative impact on technology identity of grassroots civil servants.

Existing empirical studies have provided theoretical support for the above logical chain. When the digital divide continues to widen and some grassroots civil servants are at a disadvantage in technology application due to insufficient digital skills, their recognition of digital technology will be systematically eroded (72). When the digital skill level of grassroots civil servants is difficult to match the digital technology operation ability required by their jobs, frequent setbacks in technology use will occur. This means that when individuals struggle to effectively meet the requirements of technology operation due to insufficient digital skills, their acceptance of technology will inevitably decrease, thereby weakening the value recognition and effectiveness perception of technological tools (73). Secondly, the digital divide also weakens the sense of belonging identity to technology through the tension of “participation-deprivation.” When grassroots civil servants find it difficult to fully participate in the digital decision-making process and effectively express their opinions in the digital space due to insufficient digital skills, their role identity and sense of belonging as governance subjects will inevitably be damaged (74). At the same time, the existence of the digital divide makes it difficult for some grassroots civil servants to equally access digital resources and participate in digital governance, and their marginalized experience in technology application will arouse doubts about the fairness and legitimacy of digital technology. Thus, the following research hypothesis can be proposed:

*H5:* The digital divide has a significant negative impact on grassroots civil servants’ technology identity.

As digital overload continues to intensify, grassroots civil servants struggle to cope with the multiple pressures of information overload, communication overload, and system overload, which will systematically erode their identification with digital technology. Digital overload can diminish the affective identification with technology through the continuous depletion of cognitive resources (75). According to Conservation of Resources Theory, when job demands constantly consume an individual’s limited physical and psychological resources without sufficient resource replenishment, stress responses and emotional exhaustion will occur (76). When grassroots civil servants continuously experience frustration and exhaustion under digital overload, technology is reduced from an internalizable partner to a physically and mentally draining burden, inevitably undermining the affective foundation of technology identification.

Meanwhile, digital overload also triggers a crisis of value identification with technology by creating a sense of divergence between technological tools and their intended missions (77). When grassroots civil servants repeatedly perceive that digital technology fails to serve their core work missions and instead becomes an alien force encroaching on their substantive working time, technology is transformed from a helper into an adversary, shaking the value foundation of technology identification (78). More importantly, when digital overload exceeds an individual’s coping capacity, frequent frustrations in technology use will arise, further reinforcing the negative perception that “technology is difficult to use” (79).

In summary, when information overload, communication overload, and system overload exceed an individual’s coping capacity and interact with a pressure-driven institutional context, individuals can hardly develop positive acceptance and in-depth internalization of technological tools amid sustained physical and psychological strain. As a result, the psychological foundation of technology identification will inevitably be eroded continuously. Accordingly, the following research hypothesis is proposed:

*H6:* Digital overload has a significant negative impact on the technology identity of grassroots civil servants.

#### 3.2.3 The relationship between work stress, technology identity and digital burnout

Based on the Stressor-Strain-Outcome framework, work stress, as a direct psychological response of individuals to external stressors, will eventually lead to negative work outcomes such as burnout and turnover intention when accumulated continuously (80). This finding reveals the internal mechanism by which work stress induces burnout through the sustained depletion of emotional resources. When grassroots civil servants are constantly exposed to high-intensity stress in the digital work environment, their emotional resources are continuously consumed, eventually leading to emotional exhaustion, which constitutes one of the core dimensions of digital burnout.

Conservation of Resources Theory provides deeper theoretical support for this logical chain. The theory holds that individuals are motivated to preserve existing resources and acquire new ones, and stress responses will arise when confronted with actual or threatened resource loss (81). The continuous depletion of temporal resources, energy resources and emotional resources caused by work stress ultimately results in burnout. The Job Demands-Resources Model also offers an integrative explanatory framework for the relationship between work stress and burnout. This model states that excessive job demands will continuously consume individuals’ physical and psychological resources, initiate the health impairment process, and ultimately lead to burnout (82).

In summary, as a persistent state of physical and mental strain experienced by individuals in the digital work environment, work stress systematically elevates the level of digital burnout among grassroots civil servants through multiple pathways, including the depletion of emotional resources, the weakening of work engagement, and the erosion of technology identification. Accordingly, the following research hypothesis is proposed:

*H7:* Work stress has a significant positive impact on digital burnout among grassroots civil servants.

According to Conservation of Resources Theory, individuals are motivated to preserve existing resources and acquire new ones. As a positive psychological resource, technology identification can enhance individuals’ ability to cope with job demands (81). When grassroots civil servants develop a deep identification with digital technology, technology ceases to be a stressor that depletes resources and instead becomes an instrumental resource that helps them complete work tasks and improve work efficiency.

From the perspective of specific mechanisms, technology identification inhibits the emergence of digital burnout through multiple pathways. Technology identification can reduce the risk of emotional exhaustion by strengthening technological self-efficacy (37). When grassroots civil servants hold a positive attitude toward technology, their perceived digital burden decreases significantly, which means that the consumption of emotional resources is alleviated and the risk of emotional exhaustion declines accordingly. Meanwhile, technology identification can also resist depersonalization by enhancing a sense of identity belonging. When individuals achieve value identification and gain identity belonging through technology use, technology becomes a positive force that strengthens professional identity, thereby effectively preventing the emergence of depersonalization. In addition, technology identification maintains the level of personal accomplishment by enhancing the sense of work meaning (83). As a positive psychological resource, technology identification strengthens individuals’ perceived meaning and engagement in work. When grassroots civil servants regard digital technology as an effective tool to fulfill their work mission, they are more likely to achieve a sense of accomplishment and value realization in technology application, and the dimension of “reduced personal accomplishment” in digital burnout is effectively suppressed (34).

Therefore, as a positive psychological representation of individual-technology interaction, technology identification systematically inhibits the development of digital burnout among grassroots civil servants through multiple pathways: enhancing technological self-efficacy, strengthening identity belonging, and elevating the sense of work meaning. When grassroots civil servants form deep value recognition, emotional acceptance, and behavioral integration with digital technology, technology is transformed from a resource-depleting stressor into a resource-supplementing instrumental asset, and burnout symptoms such as emotional exhaustion, depersonalization, and reduced personal accomplishment are effectively alleviated. Accordingly, the following research hypothesis is proposed:

*H8:* Technology identification has a significant negative impact on digital burnout among grassroots civil servants.

#### 3.2.4 The moderating role of leadership care and organizational incentives

Digital mismatch refers to the incongruity between grassroots civil servants’ digital skills and the digital requirements of their positions. When civil servants experience a high level of digital mismatch, they have to invest extra cognitive resources to cope with unfamiliar digital systems. According to Conservation of Resources Theory, this process continuously depletes individuals’ valuable limited resources such as self-efficacy and sense of work control. When resource loss is not compensated, a resource loss spiral will be triggered, thereby inducing significant job stress (65).

Leader care and organizational Incentives, as critical external job resources, play an important buffering role in this resource depletion process. In line with the Job Demands-Resources Model, job resources can effectively buffer the negative impacts of job demands on individuals (53, 54); Conservation of Resources Theory further reveals that individuals with abundant external resources possess stronger resilience when confronted with resource threats (76). Specifically, when grassroots civil servants suffer from resource depletion due to digital mismatch, the emotional encouragement from leaders and substantial resources such as organizational technical training and equipment support can, on the one hand, directly compensate for the lost psychological and functional resources and alleviate the net degree of overall resource depletion. On the other hand, they can enhance civil servants’ self-efficacy and organizational belonging through the resource gain effect, prompting them to reappraise digital mismatch (81). Such cognitive reappraisal significantly reduces the subjective perception of mismatch. Therefore, based on the resource buffering effect of the Job Demands-Resources Model and the resource gain mechanism of Conservation of Resources Theory, the following research hypothesis is proposed:

*H9:* Leader care and organizational incentives play a significant negative moderating role between digital mismatch (digital suspension, digital divide, digital overload) and work stress among grassroots civil servants.

The essence of digital mismatch is the structural dislocation between grassroots civil servants’ digital capabilities, technical supply, and job requirements. It will trigger technical anxiety, resistance to use, and value suspicion, thereby weakening technical identity. Existing studies have shown that problems such as system design being divorced from reality, mismatched supply and demand of skill training, and uneven digital capabilities are prevalent in grassroots digital governance (84, 85). These problems lead civil servants to regard government digital systems as an administrative burden, reduce their perception of the usefulness and ease of use of technology, and make it difficult to form a stable technical identity.

From the perspective of the moderating mechanism of leadership care, leadership care is reflected in emotional care, technical guidance, and problem response to subordinates’ difficulties in digital adaptation, which is the core content of high-quality Leader-Member Exchange (LMX) (86). In the context of digital mismatch, leadership care can convey the organization’s positive expectations for technology use, alleviate the psychological pressure of individuals caused by capability gaps or system discomfort, and help civil servants rebuild confidence in technology use (87). Based on the Leader-Member Exchange Theory, high-care leaders can reduce the negative cognition caused by digital mismatch through personalized support, strengthen individuals’ recognition of technical value, and thus positively enhance the positive tendency of transforming digital mismatch into technical identity (88).

From the perspective of the moderating mechanism of organizational support, organizational support is reflected in institutional guarantees, resource supply, training empowerment, and error-tolerance mechanisms, which are typical job resource elements (89). The Organizational Support Theory points out that employees’ perceived organizational support will improve their self-efficacy and role adaptability (90). In the scenario of digital mismatch, adequate organizational support can make up for capability shortcomings, optimize technical adaptation conditions, and reduce the inhibitory effect of digital mismatch on technical identity. Existing studies on digital transformation have confirmed that organizational support can significantly buffer the negative effects caused by technical mismatch and improve employees’ technology acceptance and recognition level (91).

Based on the above theories and literature logic, leadership care and organizational support, as key contextual resources, can effectively alleviate the negative impact of digital mismatch and strengthen grassroots civil servants’ acceptance and recognition of digital technology. Therefore, the following research hypothesis is proposed:

*H10:* Leader care and organizational incentives have a significant positive moderating effect on the relationship between digital mismatch (digital suspension, digital divide, digital overload) and technical identity of grassroots civil servants.

## 4 Methodology

### 4.1 Data collection and sample

This study primarily collected data through an online questionnaire, employing convenience sampling and snowball sampling methods, over a period of nearly three months. Researchers first selected one representative city each from eastern, central, and western China: Shanghai, Wuhan, and Chengdu. After obtaining approval from local authorities, we invited local grassroots public servants to participate in an anonymous questionnaire survey. While commissioning local authorities to assist in disseminating the survey, we also encouraged participants who completed the questionnaire to invite their colleagues to participate in the survey. All participants had experience using government digital platforms or tools in their daily work and held certain opinions.

A total of 740 questionnaires were distributed for this study. After excluding invalid questionnaires, 656 valid questionnaires remained, resulting in a response rate of 88.65%. Among these, 234 were male samples (35.67%) and 422 were female samples (64.33%). Among the samples, 328 (50%) had less than 5 years of work experience, 220 (33.54%) had 6 to 10 years of work experience, and 108 (16.46%) had over 10 years of work experience (as shown in Table 1).

**Table 1:** Description of the sample characteristic distribution.

Variables	Category	Frequency	Percentage (%)
Gender	Male	234	35.67%
	Female	422	64.33%
Age	25 or below	158	24.08%
	26–30	278	42.38%
	31–35	142	21.65%
	36–40	54	8.23%
	41 or above	24	3.66%
Education level	High school or below	14	2.13%
	College or University	434	66.16%
	Master’s degree or above	208	31.71%
Working seniority	5 or below	328	50%
	6–10	220	33.54%
	11–20	94	14.33%
	20 or above	14	2.13%

### 4.2 Variables and measures

The research questionnaire consisted of five sections. The first section collected demographic information using multiple-choice questions, including gender, age, educational level, and work seniority. All subsequent sections utilized a seven-point Likert scale for participant responses.

The second section integrated the Maslach Burnout Inventory-General Survey (MBI-GS) with the Digital Burnout Scale (DBS) developed by Maslach et al. (16) and Erten and Özdemir (19). Adapted to reflect the characteristics of digital burnout among grassroots public servants, the revised scale comprises three dimensions (behavioral burnout, emotional burnout, and meaning burnout) and six items, which are: “the application of digital technology has diminished my interest in my work,” “the application of digital technology has reduced my enthusiasm for my work,” “the application of digital technology in my work sometimes leaves me physically and mentally exhausted,” “the application of digital technology in my work sometimes makes me feel anxious,” and “I feel that the digital work I do is meaningless, and I feel that the digital work I do is worthless.” We achieved cross-context adaptation of the items through conceptual mapping: We merged the “digital disconnection” from traditional digital burnout with the “depersonalization” from occupational burnout to create items for behavioral burnout. We directly adapted the “emotional exhaustion” from occupational burnout and the “emotional depletion” from traditional digital burnout to form items for emotional burnout. Furthermore, we combined the “reduced personal accomplishment” from occupational burnout with the sense of value loss found in the “digital deprivation” of traditional digital burnout to develop new items for meaning burnout. In terms of Basis, this adaptation is grounded in solid theoretical support—it inherits the mature framework of the multidimensional model of occupational burnout and incorporates Self-Determination Theory to emphasize “meaning” as a core psychological need. It also has strong practical justification—it accurately captures the complex burnout experience of modern individuals in digital life: the desire to escape on a behavioral level, being drained on an emotional level, and feeling a sense of emptiness on a meaning level. In terms of Rationale, this approach not only ensures the measurement tool builds upon prior work (by continuing valid items) but also innovates structurally. It addresses the lack of attention given to “sense of meaning” in traditional measurements, enabling the new scale to reveal the complex nature of digital burnout more comprehensively and profoundly. This, in turn, provides a more precise assessment framework for understanding and intervening in burnout in the digital age.

The third section measured Digital Mismatch, comprising three variables: Digital Suspension, Digital Divide, and Digital Overload. Digital Suspension was measured across three dimensions: functional design, resource integration and operating mechanism, three items were: “The functions developed on digital platforms do not meet actual needs,” “the resources supporting the operation of digital platforms are insufficient” and “the operational mechanisms of digital platforms do not align with practical work requirements.” Digital Divide was evaluated through three dimensions: learning ability, knowledge level, and learning attitude, three items included: “I cannot quickly master how to use digital platforms,” “I have limited knowledge of digital technology” and “learning digital technology does not provide any substantial help for work.” Digital Overload was measured based on three dimensions: work context, information processing and performance evaluation, with three items such as: “the information processing procedures on digital platforms are overly cumbersome,” “digital platforms excessive reporting, processing, and clocking-in tasks” and “digital platforms involve excessive performance metrics”.

The fourth section measured two variables: Work Stress and Technology Identity. Work Stress was assessed using the Occupational Stress Index (OSI) developed by Cooper et al. (38), covering three dimensions: work factors, role stress, and career development, three items were: “The complexity of digital technology has increased my workload,” “the introduction of digital technology has made my job role feel ambiguous” and “the advancement of digital technology has left me uncertain about my career prospects.” Technology Identity was measured based on three dimensions: emotional identity, behavioral identity and value identity, three items included: “I enjoy working with digital technology,” “I prioritize the use of digital technology to find work solutions” and “I believe digital technology is essential for individuals to adapt to future work”.

The final section measured Leadership Care and Organizational Incentive. Leadership Care was assessed through three dimensions: emotional care, work support, and respectful inclusion, three items were: “my leader helped me manage my emotions under work pressure,” “my leader provided me with assistance when facing with challenges at work” and “my leader respected my ideas when facing with disagreements at work.” Organizational Incentive was measured across three dimensions: resource support, skills training, and performance incentives, three items were: “the organization provides sufficient resources to support the completion of digital initiatives,” “the organization conducted training to better accomplish digital tasks” and “the organization implemented a reasonable performance incentive system to better accomplish digital tasks” (see Table 2).

**Table 2:** Measurement of variables.

Variables	Means
Digital burnout	The application of digital technology has diminished my interest in my work
	The application of digital technology has reduced my enthusiasm for my work
	The application of digital technology in my work sometimes leaves me physically and mentally exhausted
	The application of digital technology in my work sometimes makes me feel anxious
	I feel that the digital work I do is meaningless
	I feel that the digital work I do is worthless
Digital suspension	The functions developed on digital platforms do not meet actual needs
	the resources supporting the operation of digital platforms are insufficient
	the operational mechanisms of digital platforms do not align with practical work requirements
Digital divide	I cannot quickly master how to use digital platforms
	I have limited knowledge of digital technology
	learning digital technology does not provide any substantial help for work
Digital overload	The information processing procedures on digital platforms are overly cumbersome
	Digital platforms excessive reporting, processing, and clocking-in tasks
	digital platforms involve excessive performance metrics
Work stress	The complexity of digital technology has increased my workload
	The introduction of digital technology has made my job role feel ambiguous
	The advancement of digital technology has left me uncertain about my career prospects
Technology identity	I enjoy working with digital technology
	I prioritize the use of digital technology to find work solutions
	I believe digital technology is essential for individuals to adapt to future work
Leadership care	My leader helped me manage my emotions under work pressure
	My leader provided me with assistance when facing with challenges at work
	My leader respected my ideas when facing with disagreements at work
Organizational incentives	The organization provides sufficient resources to support the completion of digital initiatives
	The organization conducted training to better accomplish digital tasks
	The organization implemented a reasonable performance incentive system to better accomplish digital tasks

The constructs of conceptual model were measured with multiple items based on previous studies, and some minor modifications in the wording were made to fit the context of Chinese expression culture, all of them were measured on 7-level Likert scale. The cross-cultural adaptation of the questionnaire items for variables such as Digital Burnout, Work Stress, Leadership Care, and Organizational Incentives is a rigorous multi-stage process designed to ensure that the translated versions possess conceptual validity and measurement reliability equivalent to the original versions within the target culture. The process begins with a preparatory phase, which involves a deep understanding of the theoretical constructs of the questionnaire. The core of the process follows the Brislin model of translation: first, two independent bilingual translators perform forward translation into the Chinese version, and then an expert panel synthesizes these into a preliminary version. Subsequently, a committee composed of domain experts and methodologies compares and resolves any conceptual discrepancies and performs localized wording to ensure the items are natural and appropriate. Finally, empirical validation is conducted through a pilot test: cognitive interviews are first carried out by inviting three representative individuals to reveal their comprehension process and identify potential ambiguities, thereby assessing the quality of the questionnaire.

The descriptive statistics of all variables are presented in Table 3. It can be seen that the current Digital Burnout (4.344) of grassroots public servants is at a moderate level. Among the three dimensions of Digital Mismatch, Digital Overload received the highest score of 4.928, suggesting that the implementation of digital platforms has not streamlined workflows but instead increased the burden on grassroots public servants. Digital Divide received the lowest score of 3.058, indicating that grassroots public servants possess generally high digital literacy and encounter relatively fewer cognitive barriers when using digital tools. Digital Suspension also scored high at 4.576, reflecting notable formalism in the construction of digital platforms and a disconnect from actual work needs. The high score of 4.973 for Work Stress indicates that grassroots public servants generally have a heavy burden in their work. The high score of 4.956 for Technology Identity suggests that grassroots public servants hold a positive attitude towards the application of new technologies and exhibit a strong sense of identification with digital tools. The relatively high scores for Leadership Care (4.621) and Organizational Incentives (4.251) demonstrate that caring leadership is prevalent and that incentive measures are relatively effective in providing both material and moral support.

**Table 3:** Descriptive statistical results.

Variables	Means	SD
Digital burnout	4.344	1.201
Digital suspension	4.576	1.469
Digital divide	3.058	1.163
Digital overload	4.928	1.317
Work stress	4.973	1.015
Technology identity	4.956	1.074
Leadership care	4.621	1.201
Organizational incentives	4.251	1.178

### 4.3 Empirical methodology

The empirical methodology consists of three stages, Analysis of the data was conducted utilizing SPSS 23.0 and AMOS 24.0. In the first stage, techniques for assessing the reliability and validity of the latent constructs used in the study were applied to ensure accuracy and appropriateness for measuring the intended constructs. The second stage involved assessing the structural research model using the structural equation modelling technique. The third stage examines the authenticity of the moderating effect.

## 5 Results

### 5.1 Measurement model

Before empirically testing structural model. In this study, all main constructs were measured using scales, so testing the quality of the measurement data was an important prerequisite for ensuring the validity of subsequent analyses. The results of reliability and validity analyses are shown in Table 4. The Cronbach’s *α* for all dimensions exceeded the threshold of 0.7, The CITCs of all items satisfied the general recommended level of 0.70, indicating good internal consistency reliability of the scales. Furthermore, AVE values of all dimensions were greater than 0.5, and CR values all exceeded 0.7, thus supporting the good convergent validity of the measurement model.

**Table 4:** Reliability and validity.

Items	Factor loadings	CITC	Cronbach’s α	CR	AVE
Digital burnout					
DB1	0.703***	0.789	0.887	0.891	0.577
DB2	0.628***	0.704			
DB3	0.827***	0.751			
DB4	0.818***	0.795			
DB5	0.782***	0.809			
DB6	0.781***	0.991			
Digital suspension					
DS1	0.838***	0.789	0.877	0.874	0.699
DS2	0.813***	0.798			
DS3	0.856***	0.788			
Digital divide					
DD1	0.711***	0.718	0.808	0.793	0.566
DD2	0.732***	0.776			
DD3	0.891***	0.752			
Digital overload					
DO1	0.767***	0.713	0.843	0.843	0.642
DO2	0.828***	0.736			
DO3	0.807***	0.746			
Work stress					
WS1	0.582***	0.713	0.752	0.758	0.515
WS2	0.755***	0.737			
WS3	0.798***	0.722			
Technology identity					
TI1	0.889***	0.782	0.898	0.903	0.757
TI2	0.932***	0.788			
TI3	0.783***	0.783			
Leadership care					
LC1	0.885***	0.827	0.92	0.921	0.795
LC2	0.904***	0.858			
LC3	0.885***	0.835			
Organizational incentives					
OI1	0.789***	0.745	0.834	0.836	0.629
OI2	0.806***	0.734			
OI3	0.785***	0.793			

*** *p* < 0.001.

### 5.2 Structural model

As shown in Table 5, the model fit indices are as follows: the CMIN / DF value is 2.86, which falls within the recommended threshold range of 1 to 3, indicating an acceptable model fit. The RMSEA value is 0.062, below the stringent criterion of 0.08. the goodness of fit measures for the overall confirmatory model indicated GFI (0.903), CFI (0.919), AGFI (0.906), TLI (0.908), NFI (0.911) were also over the threshold 0.9, the PGFI (0.502), PNFI (0.513), PCFI (0.524) were also over the threshold 0.5, the SRMR (0.562) has not exceeded 0.08. Thus, the findings indicate that the conceptual model satisfactorily fits the data.

**Table 5:** The overall fit coefficient.

Index		Threshold	Acceptance
CMIN/DF	2.86	<5.0	Passed
RMSEA	0.062	<0.08	Passed
GFI	0.903	>0.9	Passed
PGFI	0.502	>0.5	Passed
AGFI	0.906	>0.9	Passed
TLI	0.908	>0.9	Passed
CFI	0.919	>0.9	Passed
NFI	0.908	>0.9	Passed
PNFI	0.513	>0.5	Passed
PCFI	0.524	>0.5	Passed
SRMR	0.048	<0.08	Passed

**Figure 2:**
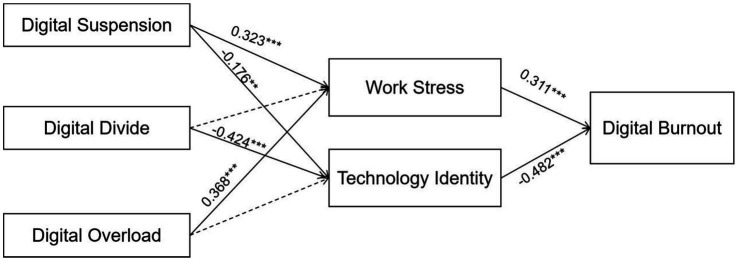
Model significant path coefficient.

The theoretical model was tested using AMOS software. The analysis results indicate that six out of the eight path coefficients in the model are statistically significant, demonstrating significant intrinsic relationships among the variables and revealing key factors influencing the level of Digital Burnout among grassroots public servants. Specific results are presented in Table 6 and Figure 2.

**Table 6:** Results of structural equation modeling.

No	Causal relationships	Estimate	S.E.	C.R.	*P*	Supported
H1	WS←DS	0.323	0.039	4.632	0.000***	Yes
H2	WS←DD	0.127	0.039	1.942	0.052	No
H3	WS←DO	0.368	0.04	5.001	0.000***	Yes
H4	DB←WS	0.311	0.114	4.607	0.000***	Yes
H5	TI←DS	−0.176	0.051	−3.072	0.005**	Yes
H6	TI←DD	−0.424	0.059	−6.658	0.000***	Yes
H7	TI←DO	−0.05	0.048	−0.876	0.381	No
H8	DB←TI	−0.482	0.067	−7.831	0.000***	Yes

* *p* < 0.05; ** *p* < 0.01; *** *p* < 0.001. C.R indicates critical ratio.

The results indicated that Digital Suspension had a significant positive effect on Work Stress (*β* = 0.323, *p* < 0.001), and H1 was supported. This implies that the greater the mismatch between the technological functions and the organizational demands, the higher the Work Stress levels among grassroots public servants. Similarly, Digital Overload also significantly and positively influenced Work Stress (β = 0.368, *p* < 0.001), and H3 was supported. This reflects that the greater the mismatch between organizational demands and individual capabilities during digital transformation, the greater the work stress experienced by grassroots public servants. A possible explanation is that grassroots work inherently involves complexity, variability, and flexibility, while the structured and linear nature of digital platforms struggles to adapt to these dynamic demands. This contradiction may trigger phenomena such as multiple information inputs and redundant reporting, collectively intensifying work stress. However, the path between the Digital Divide and Work Stress was not significant, failing to support H2. We speculate that its non-significance might be because the digital divide represents a more chronic, resource-related stressor, whereas work stress in our model may be more acutely triggered by immediate operational interruptions (digital suspension) and overwhelming demands (digital overload). In the specific context of Chinese grassroots public service, where standardized procedures are emphasized, a lack of digital skills might not directly increase daily task pressure in the same way.

Digital suspension had a significant negative effect on Technology Identity (*β* = −0.176, *p* < 0.01), supporting H5. This indicates that the greater the mismatch between the technological functions and the organizational demands, the lower the technology identity among grassroots public servants. Similarly, the Digital Divide also exerted a significant negative effect on Technology Identity (β = −0.424, *p* < 0.001), supporting H6. This implies that the greater the gap between the technological functions and the individual capabilities, the lower the technology identity level among grassroots public servants. A potential mechanism lies in the fact that grassroots public servants’ skepticism about the practicality of digital technology weakens their identity, while strong digital literacy serves as a foundation for full acceptance and effective use of digital tools. However, the relationship between Digital Overload and Technology Identity was not significant, and H7 was not supported. Because digital overload represents a tool-rational assessment of the enabling effects of digital technology in the workplace, whereas technological identification constitutes a value judgment on technology itself. Consequently, digital overload is more often viewed as a “friction at the usage level,” while technological identification is grounded in a deeper “belief at the value level.” It is therefore reasonable that the two exhibit a non-significant influence relationship.

Work Stress exerted a significant positive influence on Digital Burnout (*β* = 0.311, *p* < 0.001), confirming H4. This indicates that the higher the work stress on grassroots public servants in digital work environments, the greater their levels of digital burnout. The underlying mechanism likely involves stress depleting cognitive and emotional resources, triggering resistance toward digital technologies. This resistance fosters the perception that “digital tools increase burdens,” ultimately leading to burnout. Technology Identity exerted a significant negative effect on Digital Burnout (β = −0.482, *p* < 0.001), confirming H8. This indicates that the higher the level of technology identity among grassroots public servants in digital work environments, the lower their levels of digital burnout. The underlying mechanism may involve heightened self-efficacy in addressing digital challenges, coupled with the resolution of meaning conflicts and altered perceptions, thereby interrupting the progression from mismatch to burnout.

### 5.3 Moderating effect

To examine moderating effects, this study employed hierarchical regression analysis, incorporating interaction terms to assess the moderating effects of Leadership Care and Organizational Incentives. A significant ΔR^2^ after adding the interaction term would indicate the presence of a moderating effect. In the earlier structural equation model analysis, the paths linking Digital Suspension to Work Stress, Digital Overload to Work Stress, Digital Suspension to Technology Identity, and Digital Divide to Technology Identity all reached significant levels. This lays the foundation for the moderating effect analysis in this study. Therefore, the moderating effects were tested specifically for the above four paths.

**Figure 3:**
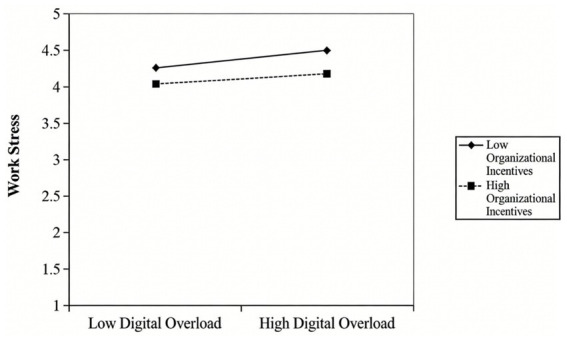
Organizational incentives moderates the influence of digital overload on work stress.

**Figure 4:**
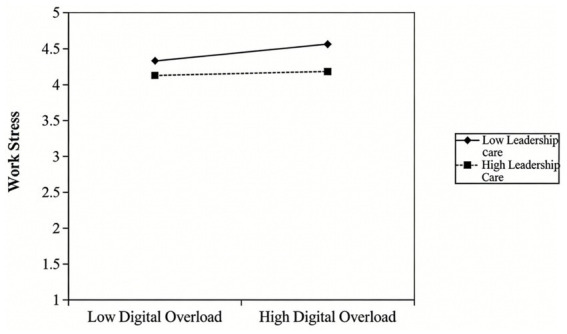
Leadership care moderates the influence of digital overload on work stress.

As shown in Tables 7–10 and Figures 3, 4, both Digital Suspension and Digital Overload exerted significant positive effects on Work Stress, while Leadership Care and Organizational Incentives exerted significant negative effects on Work Stress. After introducing the interaction terms between the moderating variables and the independent variables, it was found that the interaction term between Digital Overload and Leadership Care (*β* = −0.244, *p* < 0.001; ΔR^2^ = 0.023, *p* < 0.001) as well as that between Digital Overload and Organizational Incentives (β = −0.206, *p* < 0.001; ΔR^2^ = 0.017, *p* < 0.001) were both significantly negative. This indicates that both Leadership Care and Organizational Incentives exert a negative moderating effect on the influence of Digital Overload on Work Stress. This implies that high levels of Leadership Care and Organizational Incentives effectively buffer the negative impact of Digital Overload on Work Stress. However, within the Technology Identity mechanisms, the moderating effects of Leadership Care and Organizational Incentive were not significant. This may stem from their greater suitability for alleviating acute stress arising from workload, rather than directly addressing deeper systemic issues such as technological mismatch (Digital Suspension) or individual skill gaps (Digital Divide).

**Table 7:** The moderating effect.

Dependent variable	Work stress		Technology identity	
	Model 1	Model 2	Model 1	Model 2
Digital suspension	0.426***	0.312***	−0.236***	0.02
Leadership care	−0.93***	−0.175	0.242***	0.430***
Digital suspension*Leadership care		−0.137		−0.300
ΔR^2^	0.195	0.001	−0.236***	0.02

* *p* < 0.05; ** *p* < 0.01; *** *p* < 0.001.

**Table 8:** The moderating effect.

Dependent variable	Work stress		Technology identity	
	Model 5	Model 6	Model 7	Model 8
Digital overload	0.442***	0.295***	−0.370***	−0.536***
Leadership care	−0.104***	−0.239***	0.217***	0.128
Digital overload*Leadership care		−0.244***		0.180
ΔR^2^	0.215***	0.023***	0.212	0.002

* *p* < 0.05; ** *p* < 0.01; *** *p* < 0.001.

**Table 9:** the moderating effect.

Dependent variable	Work stress		Technology identity	
	Model 9	Model 10	Model 11	Model 12
Digital suspension	0.413***	0.367**	−0.233***	−0.077
Organizational incentives	−0.121**	−0.156	0.206***	0.325**
Digital suspension*Organizational incentives		0.054		−0.181
ΔR^2^	0.206	0.000		

* *p* < 0.05; ** *p* < 0.01; *** *p* < 0.001.

**Table 10:** The moderating effect.

Dependent variable	Work stress		Technology identity	
	Model 9	Model 10	Model 11	Model 12
Digital overload	0.429***	0.300***	−0.381***	−0.541***
Organizational incentives	−0.136***	−0.255***	0.206***	0.112
Digital overload*Organizational incentives		−0.206***		0.181
ΔR^2^	0.271***	0.017***	0.208	0.002

* *p* < 0.05; ** *p* < 0.01; *** *p* < 0.001.

## 6 Conclusion and discussion

This study achieved its intended objectives by thoroughly revealing the formation and intervention mechanisms of digital burnout among grassroots public servants within digital work environments. The results indicate that digital mismatch, as a central contradiction, contributes to digital burnout through three distinct forms—digital suspension, digital divide, and digital overload—via two mechanisms: work stress and technology identity. Specifically, digital suspension and digital overload exacerbate digital burnout by increasing work pressure; digital suspension and the digital divide intensify digital burnout by diminishing technology identity.

The study further reveals that organizational contextual factors play a crucial role in mitigating digital burnout. Leadership care and organizational incentives effectively buffered the positive impact of digital overload on work stress but showed no significant moderating effect on the technology identity mechanisms. This indicates that organizational support measures are more suitable for alleviating resource overload-type stress, but struggle to directly address structural mismatches between technology and organization or individual.

Based on these findings, it is recommended that organizations systematically establish a technology-organization-individual alignment framework during digital transformation, incorporating digital suspension, digital divide, and digital overload into occupational health management systems. By optimizing the alignment between technological tools and operational requirements, enhancing leadership and organizational support, and providing targeted digital training and resources, organizations can effectively reduce work stress, enhance technology identity, thereby lowering digital burnout and safeguarding employee well-being and work sustainability.

This study offers multiple theoretical contributions to the understanding of digital burnout among grassroots public servants. First, existing research has largely focused on the direct association between digital burnout and psychological variables, lacking systematic elaboration on its formation mechanisms. Based on the Stimulus-Organism-Response (S-O-R) model, this study clarifies the mechanism by which digital mismatch influences digital burnout through dual mechanisms of work stress and technology identity, providing an integrated theoretical explanation for understanding occupational mental health mechanisms in the digital context.

Second, this study advances beyond previous fragmented discussions of digital technology’s negative impacts by proposing a “technology-organization-individual” triadic model. It defines digital mismatch as a structural misalignment among these three elements and identifies three manifestations—digital suspension, digital divide, and digital overload. This approach offers a new framework for systematically analyzing structural contradictions in digital governance.

Finally, this study validates the buffering effect of organizational contextual factors (leadership care and organizational incentives) in the “digital overload–work stress” mechanisms, clarifying their intervention efficacy in resource-depletion stress. Meanwhile, it simultaneously reveals their insignificant role in the technology identity mechanisms, suggesting that enhancing technology identity requires cognitive transformation, thereby providing a theoretical basis for targeted interventions against digital burnout along distinct mechanisms. This is also consistent with the theoretical framework of Job Demands-Resources theory. According to the Job Demands-Resources theory, digital overload is typical job demands that can lead to digital burnout by continuously depleting employees’ physical and mental resources. Leadership care and organizational incentives, as key job resources, exert a significant negative moderating effect through a dual mechanism: Leadership care, as a form of social support resource, directly buffers the drain on employees’ energy caused by digital demands by providing instrumental assistance and emotional support, thereby weakening the health impairment pathway; whereas organizational incentives, as extrinsic motivational resources, stimulate employees’ initiative and resilience by linking the digital challenges to valuable rewards, thereby strengthening the motivational pathway. Together, these two types of resources effectively alter individuals’ perception of and ability to cope with digital stress, thereby significantly mitigating the positive impact of digital overload on work stress.

This study employs empirical analysis to reveal the dual-path mechanism through which digital mismatch influences digital burnout, and to validate the buffering effect of leadership care and organizational incentives within certain mechanisms. Recognizing that digital mismatch fundamentally stems from structural contradictions among technological functions, organizational demands, and individual capabilities, we propose systematic practical recommendations for mitigating digital burnout from technological, organizational, and individual dimensions.

At the technological level, efforts should focus on eliminating “digital suspension” by enhancing the alignment between digital tools and actual work requirements. Digital platform development should adhere to frontline operational needs through routine investigation and feedback mechanisms. Workflows and core pain points should serve as the primary basis for functional design, improving the practicality and usability of digital tools from the source to prevent technological disconnect or redundant development.

On the organizational level, emphasis should be placed on utilizing leadership care and organizational incentives to alleviate the pressure stemming from “digital overload.” Leaders should proactively show concern for employees’ work conditions within digital environments, providing necessary emotional and resource support to foster a supportive atmosphere. The incentive system should be improved by establishing performance evaluation mechanisms linked to digital technology application, combining material rewards with psychological recognition to enhance grassroots public servants’ willingness to use digital tools and their sense of technological identification, thereby reducing work stress.

At the individual level, targeted training should be provided to help employees overcome digital divide and enhance their digital literacy and technological adaptability. Training should balance operational skills with conceptual understanding, not only teaching the specific usage techniques of digital tools but also clarifying their relevance to governance objectives, to foster cognitive shifts and increase willingness to adopt. Additionally, establishing blended online-offline learning mechanisms that provide continuous support through diverse channels can help grassroots public servants transition from passive adaptation to active mastery, thereby effectively curbing digital burnout.

This study also has certain limitations. First, it employed convenience sampling and snowball sampling methods, selecting three cities in China as the sample sources. While this approach reduced research costs and implementation difficulties, it may affect the generalizability of the findings. Future research could enhance the universality of results by expanding the geographic scope of sampling and adopting random sampling methods. Second, the sample comprised only grassroots public servants. While this group is highly exposed to digital transformation and holds strong theoretical and practical significance for studying digital burnout, their work contexts and psychological mechanisms may differ from those of other occupational groups. It is recommended that future research include participants from various occupational backgrounds to examine the broader applicability of the findings. Third, the cross-sectional nature of the data limits the ability to establish causal relationships among variables. Subsequent research could utilize longitudinal data or experimental designs to further examine the effect mechanisms between variables. Finally, due to questionnaire length constraints, this study did not examine potential covariates such as sleep duration, work intensity, or family support, nor did it subdivide dimensions of leadership care and organizational incentives. Future research could explore these factors in greater depth to achieve a more comprehensive understanding of the mechanisms underlying digital burnout.

## Data Availability

The data that support the findings of this study are available from the corresponding author, upon reasonable request.
